# The complete mitochondrial genome of *Colasposoma dauricum auripenne* (Motschlsky, 1860) (Coleoptera: Chrysomelidae: Eumolpinae)

**DOI:** 10.1080/23802359.2021.1989329

**Published:** 2022-01-27

**Authors:** Xiaohui Li, Tiantian Ban, Yun Li, Chao Ma

**Affiliations:** a Institute of Horticulture, Guizhou Academy of Agricultural Sciences, Guiyang, China; b Institute of Biotechnology, Guizhou Academy of Agricultural Sciences, Guiyang, China

**Keywords:** Mitogenome, Eumolpinae, *Colasposoma dauricum auripenne*, phylogeny

## Abstract

The sweet potato leaf beetle *Colasposoma dauricum auripenne* (Motschlsky, 1860) (Coleoptera: Chrysomelidae: Eumolpinae) is one of the main pests in sweet potato. In this study, the complete mitochondrial genome of *Colasposoma dauricum auripenne* (Motschlsky, 1860) (Coleoptera: Chrysomelidae: Eumolpinae) has been sequenced. The mitogenome length is 15,614 bp (GenBank: MW528208), which includes 13 typical insect protein-coding genes (PCGs), 2 ribosomal RNA genes (rRNAs), and 22 transfer RNA genes (tRNAs), and it contains 78.6% A + T and 21.4% G + C. All PCGs start with ATD (ATT, ATA, ATG) codon and stop with termination codon TAA, TAG and single T. The phylogenetic tree showed that *Colasposoma* had been cluster together, and form a sister group relationship with *Pseudocolaspis*. The complete mitogenome of *C. dauricum auripenne* would help understand Chrysomelidae evolution.

Sweet potato leaf beetle *Colasposoma dauricum auripenne* (Motschlsky, 1860) (Coleoptera: Chrysomelidae: Eumolpinae) is one of the main pests in sweet potato, which is distributed south of China (Lv 1999; Zheng 2011). Both adults and larvae of *C. dauricum auripenne* eat sweet potatoes. Adults mainly eat sweet potato leaves into holes or notches. The larvae mainly gnaw on the tubers in the soil, eating the surface of the tubers into curved scars of different depths, and even eating the inside of the tubers, causing curved tunnels and affecting the expansion of the tubers. The sweet potato leaf beetle not only leads to the decrease of yield, but also makes the tubers of the parts eaten by insects bitter and black, which is not resistant to storage and edible (Liu 2011). Moreover, the feeding of *C. dauricum auripenne* can cause some diseases, such as black spot and soft rot (Liu 2011). Now, insect mitochondrial genes are widely used in reconstructing phylogenetic relationship and evolutionary classification (Huang 2021). In this study, we reported the complete mitochondrial genome of *C. dauricum auripenne* and phylogenetic analysis for the first time.

The *C. dauricum auripenne* was collected from sweet potato in Guizhou Province (E 106.006984, N 25.803515), China, in August 2020, and the total genomic DNA was extracted from a male adult. The genomic DNA and male genitalia are deposited in the Institute of Entomology, Guizhou University (GUGC-12813, contacts: Renhuai Dai, email: dmolbio@126.com). Sequencing was performed by illumina HiSeq 4000 (Berry Genomic, Beijing, China; 6GB raw data). We used *Pseudocolaspis* sp. PSE01 as a reference and used Geneious primer (v. 2020.0.5) for sequence assembly. Then, we annotated the assembled mitochondrial genome sequence by using MITOS web server with the invertebrate genetic code (Bernt et al. 2013), and verified the transfer RNA (tRNA) with tRNAscan-SE (Schattner et al. 2005).

*Colasposoma dauricum auripenne* mitogenome length is 15,614 bp (GenBank: MW528208), which contains 78.6% A + T and 21.4% G + C. The complete mitogenome of *C. dauricum auripenne* include 13 typical insect protein-coding genes (PCGs), 2 ribosomal RNA genes (rRNAs), and 22 transfer RNA genes (tRNAs). All PCGs start with ATD (ATT, ATA, ATG) codon and stop with termination codon TAA, TAG and single T. The size of tRNA ranged from 63 (*trn-E*, *trn-F*, *trn-T*) to 71 (*trn-K*), and the size of *12S* rRNA and *16S* rRNA were 745 bp and 1278bp, respectively.

Phylogenetic analysis was performed with 14 complete or nearly complete mitogenome sequences which obtained from GenBank, and *Pscaothea hilaris* (Cerambycidae: Lamiinae), *Monochamus alternatu*s (Cerambycidae: Lamiinae) and *Batocera lineolate* (Cerambycidae: Lamiinae) were selected as outgroups. Maximum likelihood (ML) phylogenetic trees were constructed with IQ-TREE using an ultrafast bootstrap approximation approach with 10,000 replicates based on first and second codons of 13 PCGs, the relationship of *C. dauricum auripenne* was reconstructed. Each PCG sequence was aligned using the MAFFT algorithm in TranslatorX (Abascal et al. 2010). The phylogenetic tree (Figure 1) showed that *Colasposoma* had been cluster together, and form a sister group relationship with *Pseudocolaspis*. PSE01, which would play a helpful role in the identification of *C. dauricum auripenne*.

**Figure 1.**
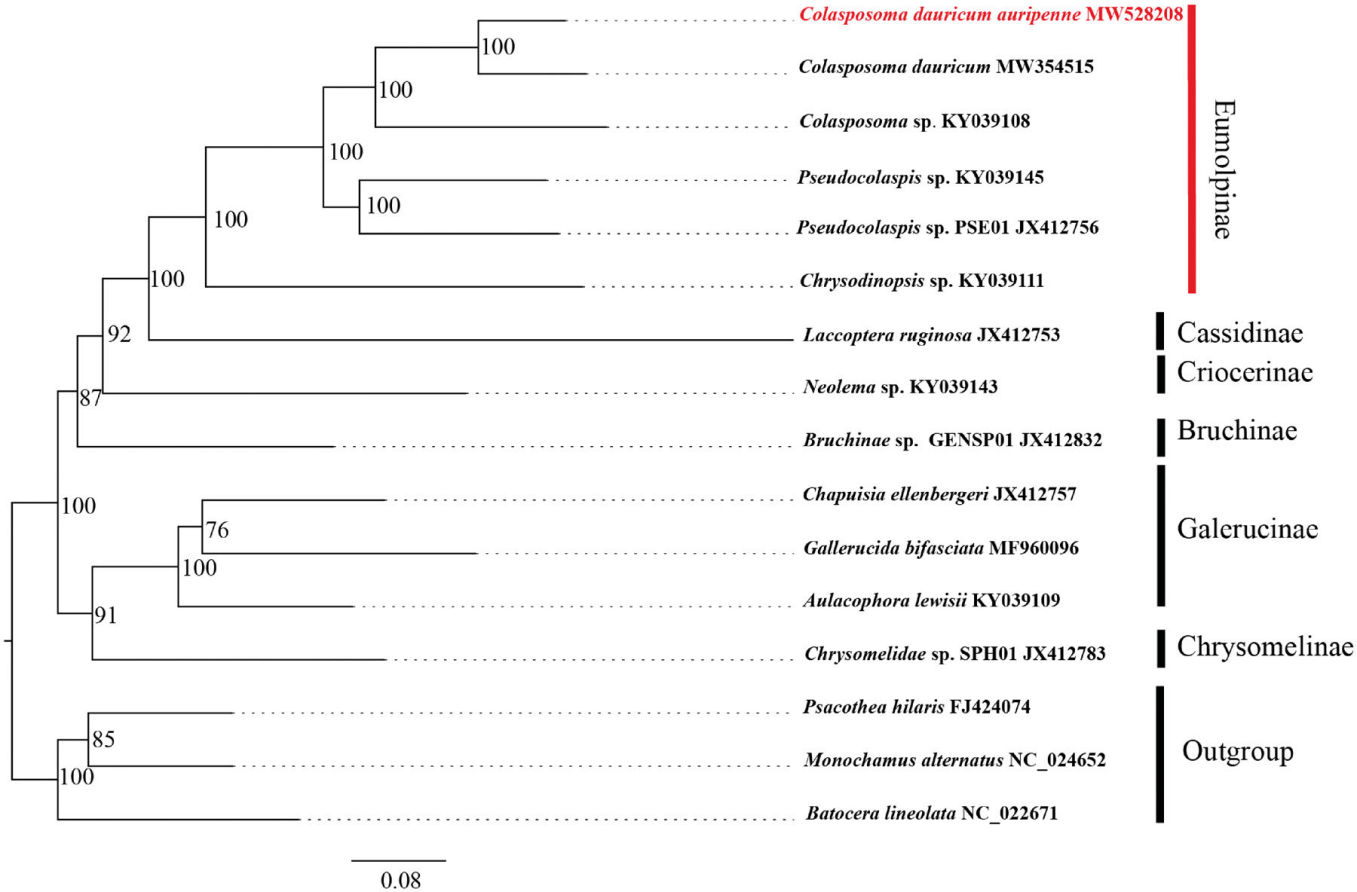
Phylogenetic analyses of *C. dauricum auripenne* (Motschlsky, 1860) based on the concatenated the first and second codon positions of 13 PCGs. The analysis was performed using IQ-TREE software. Numbers at nodes are bootstrap values. The accession number for each species is indicated after the scientific name.

## Data Availability

The genome sequence data that support the findings of this study are openly available in GenBank of NCBI at https://www.ncbi.nlm.nih.gov under the accession number MW528208. The associated BioProject, SRA, and Bio-Sample numbers are PRJNA760248, SRR15714636, and SAMN21211381 respectively.
